# Fructose-Induced Glycation End Products Promote Skin-Aging Phenotypes and Senescence Marker Expression in Human Dermal Fibroblasts

**DOI:** 10.3390/ijms26136162

**Published:** 2025-06-26

**Authors:** Antonella Rella, Dawn Layman, Rong Dang, Miriam Rafailovich, Robert Maidhof, Nadine Pernodet

**Affiliations:** 1 Research and Development, The Estée Lauder Companies, Inc., Melville, NY 11747, USA; arella@estee.com (A.R.); dlayman@estee.com (D.L.); npernode@estee.com (N.P.); 2 Materials Science & Engineering, Stony Brook University, Stony Brook, NY 11794, USA; miriam.rafailovich@stonybrook.edu; 3 Estée Lauder Research Laboratories, Melville, NY 11747, USA

**Keywords:** skin, fibroblasts, advanced glycation end products, senescence, fructose

## Abstract

Skin aging is a multi-factorial process characterized by the progressive deterioration of biomechanical properties and cellular functionality. One such factor is the formation of advanced glycation end products (AGEs), which are known to have detrimental effects on the skin, including stiffening of the extracellular matrix (ECM) and reduction of cellular proliferation. AGEs accumulate because of sugar metabolism dysfunction; however, the direct impact of elevated sugar levels on cellular physiology requires further investigation. Here, we elucidated the effects of elevated fructose levels on skin cell function using in vitro models and hypothesized that high fructose levels adversely impact cell function. By fluorescence microscopy, we observed that high fructose induced different cellularity, cell morphology, and stress fiber appearance than the controls. Skin cells exposed to high fructose levels showed impaired growth and delayed closure in an artificial wound model. Mechanistically, high fructose conditions induce inflammatory cytokines and activate the NFκB pathway. Furthermore, we observed for the first time an increase in the senescence markers *p16*, *p21*, and *p53* in response to high fructose levels. Taken together, we show that high fructose levels affect many critical skin functions that contribute to the aging process and recapitulate several aspects of aging related to AGEs.

## 1. Introduction

The skin is the largest organ of the human body and serves as the boundary between the individual and the environment. As such, the skin is subjected to both internal aging processes (senescence, altered metabolism, etc.) and external stressors (ultraviolet (UV) and visible radiation, pollution, etc.), leading to distinct structural changes in the tissue [1]. Aging is defined as the progressive accumulation of damage over time leading to predictable changes in the skin’s physical appearance, including the development of wrinkles, loss of firmness, and sagging [2]. Furthermore, aged skin has disturbed permeability, angiogenesis, lipid and sweat production, immune function, and vitamin D synthesis; manifesting as impaired wound healing, atrophy, vulnerability to external stimuli, and the development of several benign and malignant diseases [3].

Treatment of aging and aged skin requires a thorough understanding of the complex and interwoven mechanisms underlying the aging process to advance the discovery of novel therapeutic and interventional approaches. To date, multiple theories of aging have been proposed, including the theory of cellular senescence [4], decreased proliferative capacity and telomere shortening [5], mitochondrial DNA single mutations [6], and the free radical theory [7]; however, none of these fully explain all the changes observed in aging [8]. In recent years, the role of advanced glycation end products (AGEs) in skin aging has been increasingly discussed, and the potential of anti-AGE strategies has been explored [9].

As the starting material for glycation, reducing sugars such as glucose and fructose are essential for the formation of AGEs; therefore, individuals with elevated blood sugar levels, such as those with diabetes, tend to have elevated levels of AGEs in the skin [10]. Nevertheless, skin AGEs form and accumulate over time at normal physiological blood sugar levels, particularly in photo-exposed skin in both young and mature individuals [11]. The pathological implications of AGEs are ascribed to their ability to promote oxidative stress, inflammation, and apoptosis (reviewed in [12]). This signaling has been attributed to the interaction between AGEs and the Receptor for Advanced Glycation End Products (RAGE) in skin cells [13] and other cell types [14]. Furthermore, AGEs are known to play a significant role in the synthesis and degradation of skin collagen, causing shortened, thinned, and disorganized collagen fibrils that consequently reduce skin elasticity, contribute to wrinkle appearance, and delay healing (reviewed in [15]).

It has been demonstrated that rats fed a high-fructose diet for one year had significant deleterious skin alterations, including an altered collagen composition, compared to normal diet controls [16]. At the cellular level, elevated fructose levels have been associated with reduced growth kinetics of skin fibroblasts [17], which correlates with decreased cellularity observed with aging in vivo [18]. Fructose has been associated with various inflammatory conditions, including metabolic syndrome, nonalcoholic fatty liver disease, type 2 diabetes, asthma, chronic bronchitis, and arthritis; however, its pathogenic role warrants further exploration, particularly in the context of aging [19].

The aim of this study was to define the effect of glycation on skin pathophysiology in vitro. We hypothesized that exposure of human dermal fibroblasts to elevated sugar levels increases AGE levels, inducing symptoms of accelerated aging, including impaired proliferation and cell motility, altered cellular morphology, and increased inflammatory signaling. We further hypothesized that AGEs induce the expression of major cellular senescence markers (*p16*, *p21*, and *p53*) in skin dermal fibroblasts. Therefore, elevated AGE levels may serve as an interventional target for treating aged skin, which can be evaluated using our in vitro skin models.

## 2. Results

### 2.1. Human Dermal Fibroblasts Grown in the Presence of Fructose Showed Cellular Abnormalities

**Figure 1:**
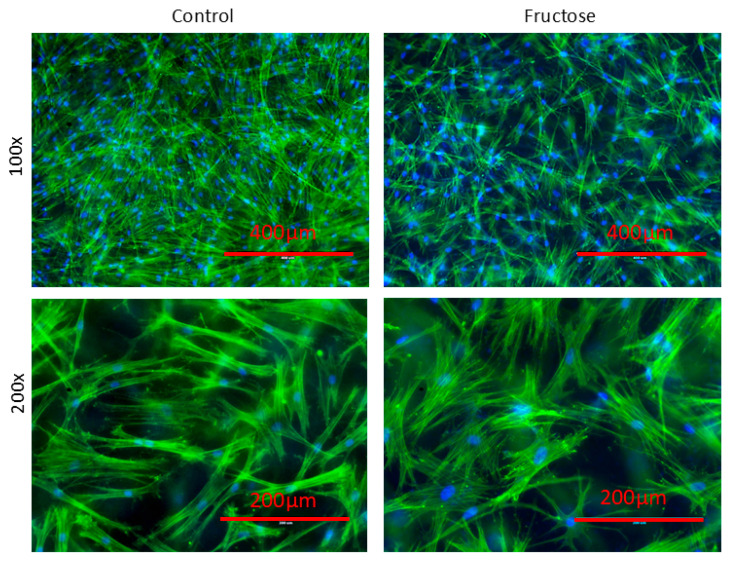
Human dermal fibroblasts grown in the presence of fructose, on a collagen gel matrix (3D culture model), showed different cellularity, cell morphology, and stress fiber appearance from fibroblasts grown in no fructose (control) culture condition. Fluorescence microscopy images of actin cytoskeleton filaments and nuclei of human fibroblasts stained with phalloidin Alexa Fluor 647 (F-actin, green) and DAPI (nuclei, blue). Human fibroblasts were cultured on a collagen gel matrix without (**left**) or in presence of fructose (**right**) acquired at low (100×, **top**) and high magnification (200×, **bottom**).

To investigate the effect of sugars on skin fibroblasts and extracellular matrix (ECM) fiber organization, we chose fructose as the main source of sugar because several in vitro studies suggest that fructose is a much more potent initiator of the Maillard reaction, whereas glucose is among the least reactive sugars [20,21]. Skin fibroblasts were grown for 14 days in the presence of fructose in a collagen gel matrix (3D culture model). We performed a preliminary investigation to evaluate the effects of various concentrations of fructose. A concentration of 20 mg/mL of fructose was chosen for all subsequent studies because it did not adversely impact the cell morphology (Supplementary Figure S1). Fluorescent labeling of the actin cytoskeleton in fibroblast-laden collagen gels cultured with fructose showed clear differences in cell density and morphology compared to the controls (Figure 1). The control constructs showed greater cellularity in micrographs than those cultured with fructose. At higher magnification, differences in cell morphology can be observed; fibroblasts in fructose-treated constructs appeared less elongated with poorly defined long and short axes compared to controls, potentially due to a lower cell density and, therefore, more space to spread. Furthermore, fructose-treated cells showed more distinctive stress fibers, which may indicate differentiation towards a myoblast-like phenotype [22].

**Figure 2:**
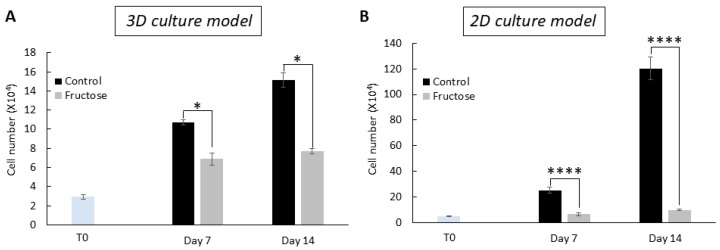
Fibroblast growth is impaired in the presence of fructose. Number of fibroblasts grown in a 3D collagen gel matrix culture model (**A**) and in a 2D cell culture model (**B**) without and with fructose over the course of 14 days. **** *p* < 0.0001, * *p* < 0.05, and ns = non-significant for control vs. fructose condition at experimental timepoint, two-way ANOVA with Tukey’s multiple comparison test. Error bars represent mean ± SD.

Quantification of the cell number showed a gradual increase in cellularity over the 14 days of culture for constructs grown with and without fructose supplementation (Figure 2A). At day 1 post-seeding, the fructose-cultured constructs had a similar cell density compared to the control (29,100 ± 2300 vs. 29,800 ± 1400, respectively, *p* = 0.67). However, fructose-cultured constructs had significantly fewer cells than the control on day 7 (68,800 ± 6200 vs. 107,100 ± 2600, respectively, *p* < 0.001) and day 14 (77,000 ± 2900 vs. 151,200 ± 7700, respectively, *p* < 0.001). Similar behavior was also observed in a 2D culture model (Figure 2B), with a greater decrease in cell number compared to the control compared to the 3D model. Taken together, our findings confirm that fructose has a significant inhibitory effect on the proliferation of fibroblasts in our 3D and 2D cell culture models.

### 2.2. Fructose Increases the Production of Advanced Glycation End-Products

**Figure 3:**
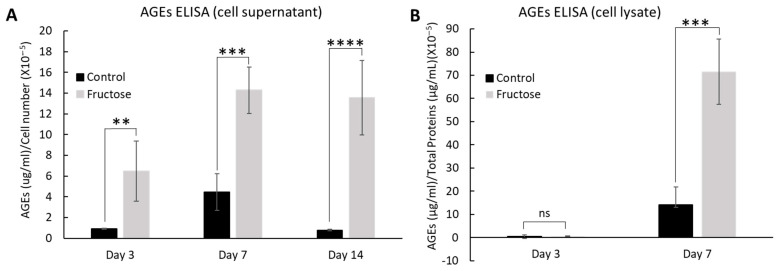
Fructose treatment increases AGEs in a 2D cell model. Culture media of adult fibroblasts treated with fructose over the course of 14 days (**A**) and proteins extracts at day 7 (**B**) showed a significant increase in AGEs. Changes of AGEs were assessed by competitive ELISA **** *p* < 0.0001, *** *p* < 0.001, ** *p* < 0.01, and ns = non-significant for control vs fructose condition at experimental timepoint, two-way ANOVA with Tukey’s multiple comparison test. Error bars represent mean ± SD.

To further investigate the detrimental effect of fructose on ECM fiber organization and cellular proliferation, we cultured skin fibroblasts in the presence of fructose for 14 days, and AGE levels were evaluated using the enzyme-linked immunosorbent assay (ELISA) technique. Culture supernatants of cells treated with fructose displayed a significant increase in AGEs starting at 3 days post-treatment (Figure 3A). An increase in AGEs was observed between days 3 and 7, whereas the AGE level plateaued between days 7 and 14. AGE levels were also evaluated in the protein fraction of skin fibroblasts grown in the presence of fructose for 7 days. Interestingly, an increase in AGEs in the cellular fraction was only observed on day 7, not on day 3 (Figure 3B). These data support our hypothesis that an increase in AGEs is potentially responsible for the abnormal skin cell phenotype.

### 2.3. Advanced Glycation End-Products and Inflammation

**Figure 4:**
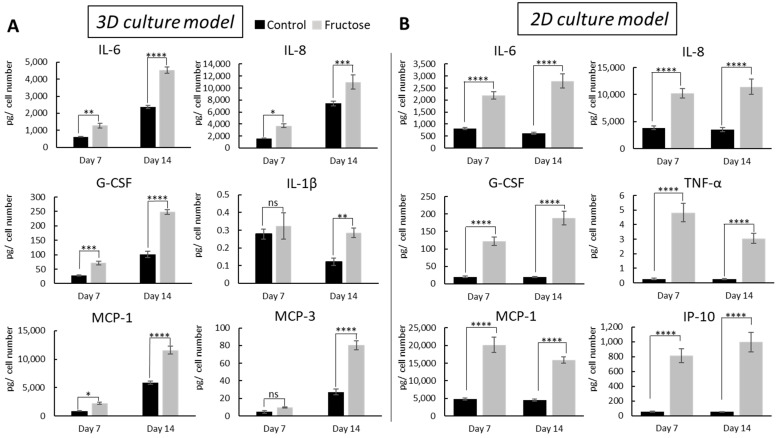
Fructose treatment increases inflammatory mediators in 3D and 2D cell in-vitro culture models. Cytokines and chemokines expression data of fibroblasts cultured in 3D collagen gels (**A**) or 2D (**B**) for 14 days with or without fructose supplementation. The expression level of cytokines and chemokines was evaluated using a Luminex magnetic beads kit. **** *p* < 0.0001, *** *p* < 0.001, ** *p* < 0.01, * *p* < 0.05, and ns = non-significant for control vs fructose condition at indicated experimental timepoint, two-way ANOVA with Tukey’s multiple comparison test. Error bars represent mean ± SD.

High-sugar exposure and AGEs have been implicated in a vicious cycle of inflammation (reviewed in [23]), so we chose to further investigate the role of AGEs in inducing inflammation in our skin cell models. We measured the cytokines and chemokines secreted by fibroblasts cultured in 3D collagen gels and 2D cultures with and without fructose for 14 days. In the 3D culture model, the levels of IL-6, IL-8, G-CSF, IL-1β, MCP-1, and MCP3 were significantly elevated in the fructose-treated group compared to the control group (Figure 4A). Similar results were observed in the 2D culture model, with a significant increase in IL-6, IL-8, G-CSF, TNF-α, MCP-1, and IP-10 levels on days 7 and 14 after fructose treatment (Figure 4B). Our findings provide evidence that fructose treatment and AGE development increase a variety of pro-inflammatory mediators in our 2D and 3D cell culture models.

**Figure 5:**
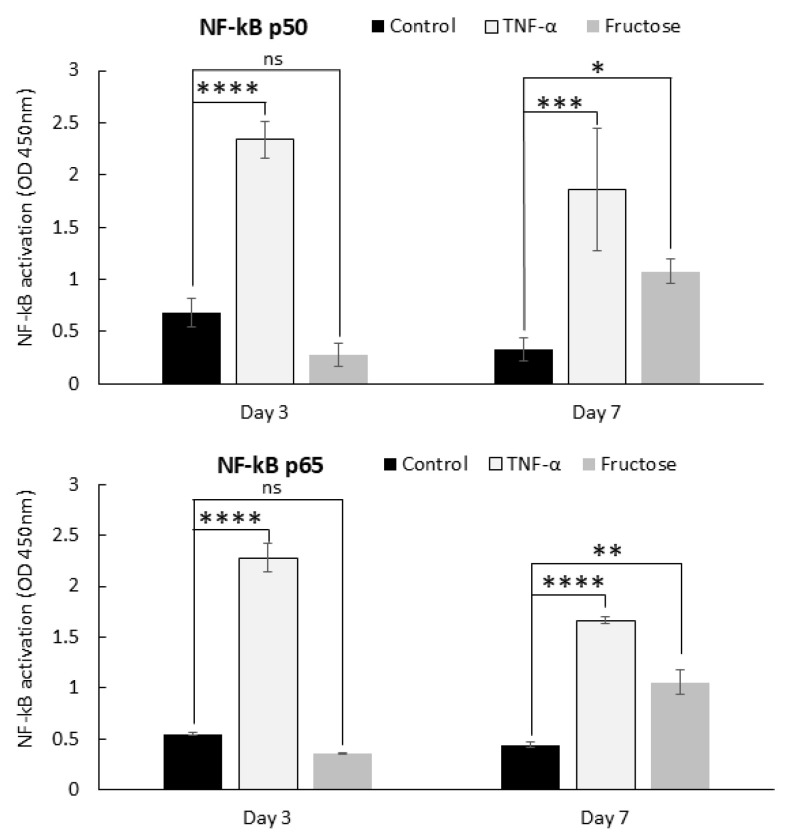
Nuclear translocation of NF-kB subunits increases in dermal fibroblasts after fructose treatment. Nuclear extracts were prepared from human dermal fibroblasts treated with TNF- α (positive control) and fructose for 3 and 7 days. The extracts were assessed for p50 and p65 using the TransAM NF-kB Family Kit. **** *p* < 0.0001, *** *p* < 0.001, ** *p* < 0.01 and * *p* < 0.05, and ns = non-significant for control vs fructose condition at each experimental timepoint, two-way ANOVA with Tukey’s multiple comparison test. Error bars represent mean ± SD.

To better understand the inflammation process in skin fibroblasts, we followed the transcription factor NF-κB, a canonical regulator of inflammation [24]. Since inflammatory cytokines increased by day 7 of fructose treatment, we chose to study an earlier timepoint (day 3) to detect whether an upstream inflammatory response had begun. We found a significantly increased level of NF-κB p50 and p65 subunits in the nuclear fraction of skin fibroblasts treated with TNF-α on days 3 and 7 (positive control) and a significant increase in subunits p50 and p65 in fibroblasts treated with fructose for 7 days (Figure 5). Therefore, our findings demonstrate that in response to fructose, latent cytoplasmic NF-κB is activated, enters the nucleus, and potentially induces the expression of inflammatory cytokines in response to cellular stress triggered by the increase in AGE level.

### 2.4. Elevated Level of Advanced Glycation End Products Delays Skin Wound Healing

**Figure 6:**
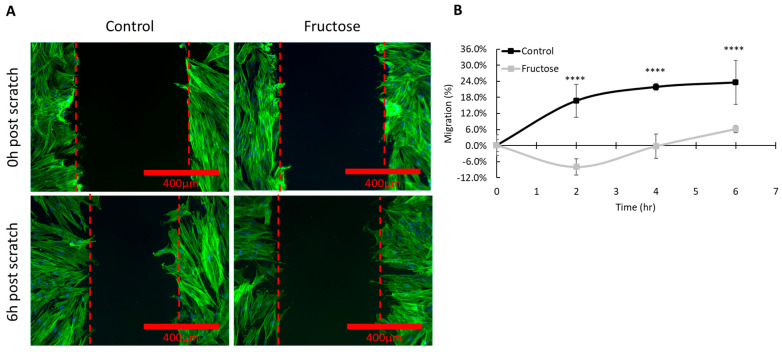
Fructose treatment alters the migration and proliferation pattern of fibroblasts. (**A**) Representative migration/proliferation assay for fibroblasts grown without (**left**) and with fructose (**right**) at 0 h and 6 h post scratch. For visualization cells were stained for actin and imaged under fluorescence. Red dotted lines indicate scratch margins during the experimental time course. (**B**) Migration was calculated using a normalized equation relative to the initial scratch width, over the course of 6 h, across replicate wells (n = 7). **** *p* < 0.0001 for control vs fructose condition at indicated timepoint, two-way ANOVA with Sidak’s multiple comparison test. Error bars represent mean ± SD.

To evaluate the effect of fructose exposure on the migration pattern of skin fibroblasts, we performed a scratch assay. In our in vitro wound healing mimic assay, we observed that fructose significantly decreased the speed of gap closure over 6 h compared to the control (Figure 6). Fibroblasts in the control group began to close the gap at the first time point measured (2 h), whereas fibroblasts in the high fructose condition had a significant increase in gap width, possibly due to changes in cell morphology (as observed in Figure 1). Although the high fructose cultured cells trended towards gap closure during the 6-h experimental observation, the gap width was not significantly decreased compared to the initial width. These findings are in agreement with several studies showing that enhanced levels of AGEs have been linked to delayed wound healing due to the possible senescent phenotype of cells necessary for the wound repair process [25].

### 2.5. Advanced Glycation End Products and Cellular Senescence

**Figure 7:**
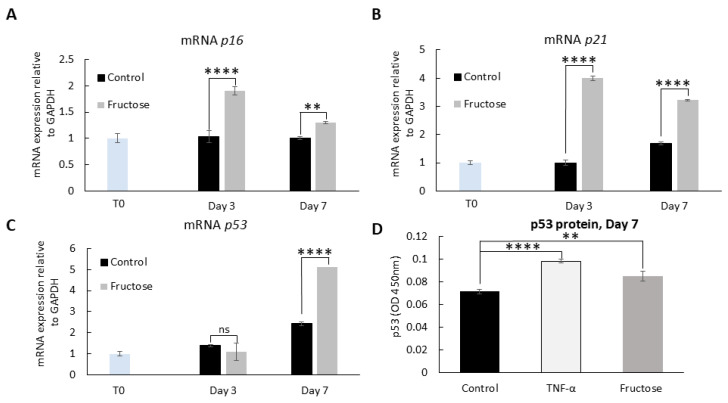
Major markers of cellular senescence increase after treating human dermal fibroblasts with fructose. (**A**–**C**) Fructose treatment in adult NHDF leads to an increase of *p16*, *p21* and *p53* mRNA, assayed by qRT-PCR. (**D**) Whole protein extracts were prepared from human dermal fibroblasts treated with fructose for 7 days. The extracts were analyzed with Human p53 ELISA Kit. qRT-PCR, quantitative Real-Time Polymerase Chain Reaction.**** *p* < 0.0001, ** *p* < 0.01, and ns = non-significant for control vs fructose condition at experimental timepoint, two-way ANOVA with Tukey’s multiple comparison test. Error bars represent mean ± SD.

Since we previously observed that fructose reduces cellular proliferation (Figure 2), we investigated the effect of high sugar on the activation of *p16*, *p21*, and *p53*, which play a critical role in cellular responses to stress, leading to cell growth arrest and cellular senescence [26,27]. Induction of *p16*, *p21*, and *p53* was examined in skin fibroblasts treated with and without fructose over 7 days. Fibroblasts treated with high fructose significantly increased the expression of *p16*, *p21*, and *p53* at the transcriptional level (Figure 7A–C) and p53 at the translational level (Figure 7D) in fibroblasts compared to the control. These findings suggest a direct correlation between AGEs and cellular senescence.

## 3. Discussion

Biochemical changes that underlie skin aging are multi-faceted and glycation of skin proteins is one such change that correlates with visible aging. The accumulation of glycated proteins is known to increase matrix stiffness [28], cause skin yellowing [29], and have negative functional effects on skin cell behavior, such as reduced proliferation [30], reduced wound healing capability [31], increased senescence [32], and increased inflammation [23]. Therefore, glycation has become an attractive target for biological interventions to reduce the visible signs of skin aging. In this study, for the first time, we focused on the role of elevated sugar levels in the functional properties of normal skin cells and showed that these changes coincide with a robust inflammatory and pro-senescent response in vitro.

The biosynthesis of glycated proteins is predicated on the availability of reducing sugars that undergo a variety of intermediate pathways to react with proteins and form advanced glycation end products (reviewed in [33]). Elevated sugar levels in patients with metabolic disorders, such as diabetes, are well known to correlate with elevated AGEs [34], which can be detected by invasive [35] and non-invasive [36] methods in the skin. In this study, we sought to further define the effects of elevated sugar levels on skin cell function in vitro to better understand their influence on cell behavior and establish models for future screening of anti-glycation technologies.

We chose to explore both 3D and 2D cell culture models in this investigation. The 3D model provides physiologically relevant spatial and biomechanical cues that are known to influence cell growth, whereas the 2D model allows a deeper evaluation of the mechanism of action related to the effects of fructose on skin cells. Regardless of the model chosen, we observed similar findings related to growth inhibition and inflammatory mediators with fructose treatment.

We found that many features associated with skin glycation were recapitulated in our in vitro high-sugar skin models. Glycation alters the biomechanical properties of the skin, leading to increased ECM stiffness [37], as it does in other tissues, such as tendons [38] and vasculature [39]. In our experiments, we observed that in high sugar cultures, fibroblasts appeared less elongated, which might be due to the lower cell number and more space to spread, and they exhibited a more well-defined actin stress fiber appearance. Cytoskeletal proteins are important in providing biomechanical stability to the cell [40] and are crucially involved in numerous cellular functions, such as migration [41] and cellular division [42]. Previously, intermediate filaments, such as vimentin in fibroblasts and CK10 in keratinocytes, have been found to be modified by AGEs [43,44]. The altered actin stress fiber appearance we observed may be due to the inability of fibroblasts to contract the stiffened extracellular matrix, as previously suggested for fibroblasts grown on glycated collagen matrices [45].

In our in vitro model, skin fibroblasts exposed to high fructose levels showed a significant increase in AGEs in the cellular and extracellular fractions. This is likely a major cause of the tissue stiffening associated with aging.

Fibroblasts grown in 3D and 2D cultures also showed significant changes in biochemical and functional behaviors in response to high sugar concentration. Fibroblast significantly increased the secretion of the inflammatory cytokines IL-6, IL-8, G-CSF, IL-1β, TNF-α, and MCP-1/3, indicating a robust inflammatory response. Our observed cytokine changes may indicate activation of the NF-kB signaling pathway, which has been shown to be mediated by AGEs in a wide spectrum of tissues and disease states (reviewed in [46]). We found a significant increase in the nuclear translocation of the NF-kB transcription factor, as measured by its p50 and p65 subunits. Fibroblasts grown with high fructose were also less proliferative and slower to close an in vitro wound model than controls, and our findings are in agreement with in vivo observations of decreased proliferation [30] and delayed wound healing [31] in aged skin.

Previous work has shown that AGEs decrease the proliferation of fibroblasts cultured in monolayers [47] and increase markers of fibroblast senescence [48]. AGEs have also been associated with senescence-associated changes in various tissues in vivo (reviewed in [49]). Our findings show for the first time that fructose significantly upregulates the major cellular senescence players, *p16*, *p21*, and *p53*, in skin cells. One theory regarding the mechanism of skin aging is that chronic low-level inflammation and increased pro-inflammatory cytokines result in a shift towards cellular senescence, which impacts skin biochemistry and the extracellular matrix over time, resulting in an aged appearance and function [50]. Based on these results, AGEs are significant contributors to increased inflammation in the skin.

Altogether, these data support our hypothesis that the increase in AGEs due to fructose exposure induces the release of pro-inflammatory molecules and the activation of *p16*, *p21*, and *p53*, resulting in skin cellular senescence [51], which limits the proliferation of new cells and leads to impaired regeneration in aged skin. A more thorough investigation of the relationship between *p16*, *p21,* and *p53* and senescence in the context of sugar overload is beyond the scope of this preliminary investigation, but our novel findings suggest that the physiological changes we observed may be related to this regulatory network.

A limitation of this study is that we did not directly compare the effects of different reducing sugars in our models; therefore, we cannot judge the relative contributions of different sugar chemistries to the overall behavior. In terms of AGE formation, it has been suggested that fructose is about 10 times more reactive than glucose, although typical concentrations of fructose are only 1% that of glucose in blood plasma [19]. However, glucose and fructose are both reducing sugars that participate in the formation of AGEs and have clinical relevance in the context of skin aging. We cannot rule out the effects of metabolic pathways other than glycation in our in vitro models, although the precise interplay between AGEs and cell metabolism may be difficult to separate, particularly in vivo. Future studies should explore the effects of interventional strategies on our models, such as limiting oxidation (a key step in the formation of many AGEs) [52], introducing AGE inhibitors [53], or treating with ‘AGE-breakers’ to induce AGEs clearance [54].

## 4. Materials and Methods

### 4.1. Cell Cultures

Normal human dermal fibroblasts (NHDF) from healthy female donors aged 40 and 42 years were supplied by ZenBio, Inc. (Durham, NC, USA). NHDF were cultivated in Dulbecco’s Modified Eagle’s Medium (DMEM, Life Technologies Cat#119611965092, Carlsbad, CA, USA) with 10% Bovine Calf Serum and 1% penicillin/streptomycin (supplemented DMEM media) in a humidified atmosphere of 5% CO_2_ and 37 °C, and experiments were performed at approximately passage 8. For fructose treatment, NHDF were treated with 20 mg/mL fructose (Sigma Cat#F0127-500G, St. Louis, MO, USA) in supplemented DMEM. Fibroblasts were counted using a Vi-Cell XR Cell Viability Analyzer (Beckman Coulter, Indianapolis, IN, USA).

### 4.2. Fluorescence Imaging of Human Dermal Fibroblasts in Collagen Gel Matrix

A collagen gel matrix was created using the following ratios: 1 part of 47.62% 10X DMEM (Biochrom/Gentaur, Cat#F0455, San Jose, CA, USA), 4.762% L-Glutamine 200mM (Biochrom/Gentaur, Cat# K0283), and 47.62% Bovine Calf Serum (Hyclone/Cytiva, Cat#SH30072.03, Willmington, DE, USA); 8 parts of 3 mg/mL VitroCol Human Collagen (Advanced Biomatrix, Cat#5007, Carlsbad, CA, USA); 1 part of 24.62% of 7.5% Sodium Bicarbonate (Biochrom/Gentaur, Cat#L1713) and 27.76% of 1 N Sodium Hydroxide (Sigma, Cat#S2770), the remaining aqueous volume consisting of 47.62% of cell culture was suspended in 1x supplemented DMEM. The matrix solution was kept on ice (2–10 °C) to prevent gelation. Fibroblasts were inoculated at a density of 7.5 × 10^4^ cells/mL into the chilled matrix solution and rolled to mix. Chilled matrix solution (1 mL) containing fibroblasts was pipetted into Flexcell Tissue Train 6-well circular foam plates (Flexcell International, Cat#UF-4001C, Burlington, NC, USA), and the gel was guided to saturate the foam by gently compressing it with a pipette tip. To set the matrix, the Flexcell Tissue Train plates were transferred to standard cell culture incubation conditions of 5% CO_2_, 37 °C, and 95% humidity for 3 h. At the end of the 3 h, 3 mL of 1× supplemented DMEM media was added to each well. The collagen matrix with cell culture was maintained under standard cell culture conditions for the duration of the experiment.

Visualization of actin (Alexa Fluor 488 phalloidin, Invitrogen, Cat#A12379, Carlsbad, CA, USA) and DAPI (4′, 6-Diamidino-2-Phenylindole,dilacetate, Invitrogen, Cat#D3571) stained fibroblasts in the collagen gel matrix was achieved by removing the flexible membrane from the Flexcell 6-well plate with a scalpel and inverting the gel into a standard 6-well plate for imaging purposes, as the silicone membrane of the Flexcell well bottom is not optically clear for microcopy. Images were captured at 100× and 200× magnification using an AMG EVOS imaging station (AMG, Bothell, WA, USA) and the Nikon Elements BR software v3.2 (Nikon, Melville, NY, USA).

In the 3D culture model, nuclei were identified based on DAPI staining, and cell counts were quantified using the “Object Count” tool in the NIKON NIS-Elements software v3.2, following threshold-based segmentation. For each well, the average nuclear count was calculated from six representative images. To estimate the total cell number per gel, these counts were extrapolated using a unitless conversion factor that accounted for the imaged area relative to the total gel area.

### 4.3. Measurement of Advanced Glycation End Products in Protein Extracts and Culture Media

NHDF were cultured in the presence or absence of fructose, as previously described, and the cells and culture media were collected at the designated timepoints. The cells were then harvested for protein, and a colorimetric assay (Bio-Rad Protein Assay Kit, Cat#5000001, Hercules, CA, USA) based on the Bradford method was used to measure the protein concentration. Aliquots of proteins and culture media were assayed using the enzyme-linked immunosorbent assay (ELISA) technique to determine AGE levels (Abcam AGE Assay kit, Cat#ab238539, Cambridge, MA, USA) as per the manufacturer’s instructions. The absorbance was measured using a microplate reader at 450 nm.

### 4.4. Inflammatory Cytokines-Chemokines Profiling

NHDF culture supernatants were collected at the designated timepoints, and frozen at −80 °C until ready to use. The collected supernatants were assayed according to the manufacturer’s instructions using the Milliplex MAP Human Cytokine/Chemokine Magnetic Bead Panel (EMD Millipore, Cat#HCYTOMAG-60K-23, Billerica, MA, USA) kit.

### 4.5. Measurement of Activated NF-κB Subunits p50 and p65

NHDF were seeded at 2.5 × 10^5^ cells in 100 mm cell culture dishes. The cells were treated with supplemented DMEM (control) or supplemented DMEM containing fructose for the duration of the experiment (7 days). Twenty-four hours prior to harvesting, the cells were treated with 100 ng/mL TNF-α (Recombinant Human TNF-α Protein-R&D systems, Cat#210-TA, Minneapolis, MN, USA) as a positive control [55]. The cells were then harvested for protein analysis at the indicated time points. Nuclear extraction was performed using a Nuclear Extract Kit (Active Motif, Inc. Cat#40010, Carlsbad, CA, USA) according to the manufacturer’s protocol. A colorimetric assay (Bio-Rad Protein Assay Kit, Cat#5000001) based on the Bradford method was used to measure the protein concentration. Nuclear extract (10 µg) fractions were used to measure NF-κB activation in the cells. Active NF-κB subunits p50 and p65 were evaluated using the Trans AM NFκB Family Transcription Factor Assay Kit (Active Motif, Inc. Cat#40096) according to the manufacturer’s instructions. Standards were prepared from recombinant p50 (Active Motif, Inc. Cat#31301) and p65 (Active Motif, Inc. Cat#31102).

### 4.6. Cell Migration-Proliferation Assay

The cells were incubated in 60 mm dishes either with supplemented DMEM media (control) or a solution of 20 mg/mL fructose in supplemented DMEM media for 5 days. At the end of 5 days, the plates were washed with 3 mL of sterile Dulbecco’s Phosphate Buffered Saline (DPBS) and seeded with NHDF at a density of 2.25 × 10^5^ cells per dish. Cells were cultured and left undisturbed under standard cell culture conditions for 3 days.

After confluence was reached, the cultures were scratched uniformly with a 200 μL pipette tip, and each dish was subsequently fixed using 3.7% methanol-free formaldehyde at t = 0, 2, 4, and 6 h post-scratch.

Samples were permeabilized with 0.4% Triton X 100 (Fisher Cat#BP151-100, Agawam, MA, USA) and stained for actin and DAPI using the reagents described for digital imaging (20×) with the AMG EVOS microscope. Scratch width was determined using measurement tools utilizing the Nikon Elements software v3.2.

Cell migration was quantified by measuring the change in scratch width over time using a normalized equation relative to the initial scratch width. At each time point, the distance within the scratch was measured and compared with the width at 0 h. Migration (%) was calculated using the following formula:

((distance at 0 h − distance at current time point)/distance at 0 h) × 100 [56]

This approach standardizes the data to baseline measurements. Average migration was then calculated across replicates (n = 7) for each time point and condition.

### 4.7. Quantitative Real-Time PCR (qRT-PCR)

NHDF were cultured in the presence or absence of fructose for 7 days. On days 3 and 7, cells were harvested, and RNA was isolated and quantified using the miRNeasy Micro Kit (Qiagen, Cat#217084, Germantown, MD, USA) and Quant-it™ RiboGreen RNA Assay Kit (Invitrogen, Cat#R11490), respectively. RNA (1 μg) was reverse transcribed into complementary DNA (cDNA) using the SuperScript™ IV VILO™ Master Mix (Thermo Fisher, Cat#11756050, Agawam, MA, USA) or TaqMan microRNA Reverse Transcription Kit (Thermo Fisher, Cat#4366596). The resulting cDNA was amplified using a Quant Studio 7 Flex system (Applied Biosystems, Foster City, CA, USA) with a universal PCR master mix (Life Technologies, Cat# 4440040) and the recommended PCR conditions for the quantitative assessment of gene transcript levels in the samples. To assess the expression of cellular senescence markers, *p16*, *p21* and *p53*, TaqMan assay probes (Applied Biosystems), *p16* (CdKn2a, Hs00923894_m1), *p21* (Cdkn1a, Hs00355782_m1), and *p53* (Tp53, Hs01034249_m1) were used. The data were normalized to GUSB (Hs00939627_m1). The qRT-PCR data were calculated using the 2^−ΔΔCT^ method.

### 4.8. Detection of p53 Protein

NHDF were seeded and treated as described above. The cells were then harvested for protein analysis at the indicated time points. Protein levels were quantified using the Bradford assay. Whole-cell extract (5 μg) was used to detect p53 protein using the Enzyme-Linked Immunosorbent Assay Simple Step ELISA^®^ kit (Abcam, Cat#ab171571) according to the manufacturer’s instructions.

### 4.9. Statistical Analysis

All data are presented as mean ± standard deviation of 3 independent experiments, with at least 3 technical replicates unless otherwise indicated. Comparative data were analyzed using Graph-Pad Prism 10 (GraphPad Software, LLC, San Diego, CA, USA) with ANOVA and appropriate post hoc testing, as indicated in the figure legends. A *p*-value of less than 0.05 was considered statistically significant.

## Data Availability

The original contributions presented in this study are included in the article/Supplementary Materials. Further inquiries should be directed to the corresponding author.

## Supplementary Materials

The supporting information can be downloaded at: https://www.mdpi.com/article/10.3390/ijms26136162/s1.
