# Supplementary figures and images for: Fructose-Induced Glycation End Products Promote Skin-Aging Phenotypes and Senescence Marker Expression in Human Dermal Fibroblasts
Source: Int J Mol Sci. 2025 Jun 26;26(13):6162. doi: 10.3390/ijms26136162 (PMC12250180; doi:10.3390/ijms26136162)

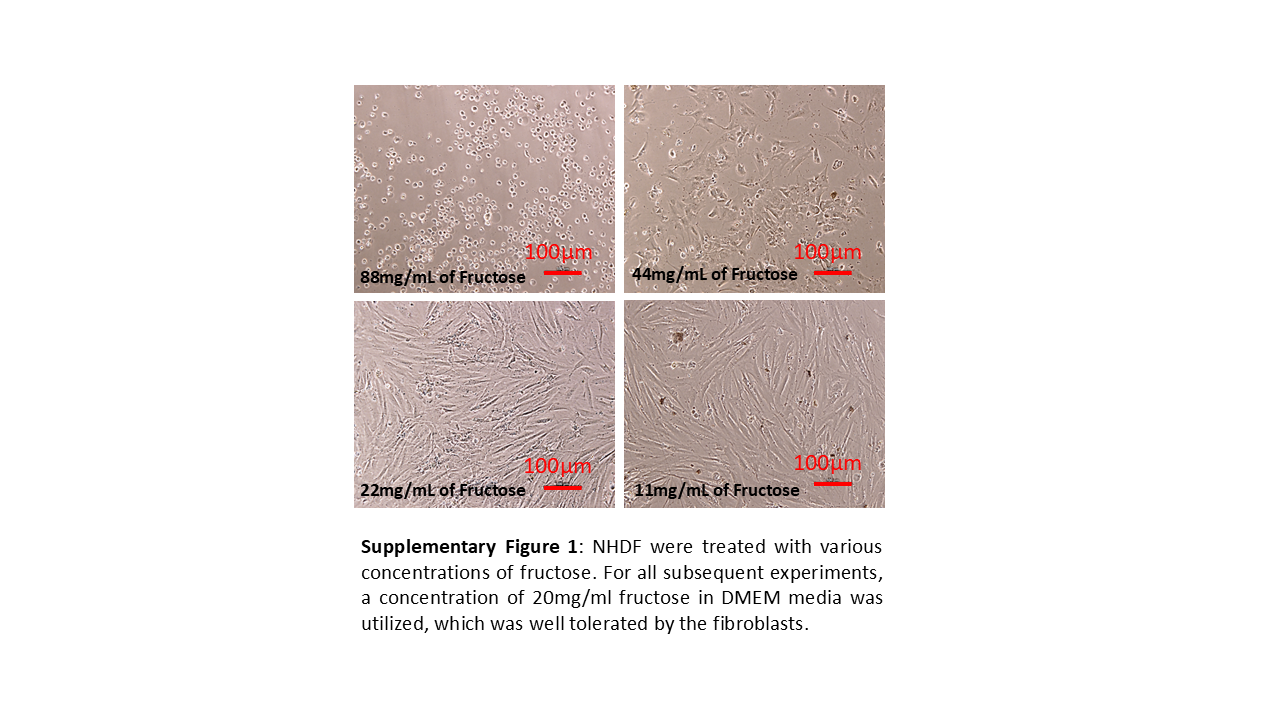

Supplement: Supplementary file 1 [file ijms-26-06162-s001.zip › ijms-3663223-supplementary.TIF]
